# Epigenetic Signatures in Arterial Hypertension: Focus on the Microvasculature

**DOI:** 10.3390/ijms24054854

**Published:** 2023-03-02

**Authors:** Alessandro Mengozzi, Sarah Costantino, Alessia Mongelli, Shafeeq A. Mohammed, Era Gorica, Valentina Delfine, Stefano Masi, Agostino Virdis, Frank Ruschitzka, Francesco Paneni

**Affiliations:** 1 Center for Translational and Experimental Cardiology (CTEC), Zurich University Hospital, University of Zurich, 8952 Schlieren, Switzerland; 2 Health Science Interdisciplinary Center, Scuola Superiore Sant’Anna, 56127 Pisa, Italy; 3 Department of Clinical and Experimental Medicine, University of Pisa, 56126 Pisa, Italy; 4 Department of Cardiology, University Heart Center, University Hospital Zurich, University of Zurich, 8091 Zurich, Switzerland; 5 Institute of Cardiovascular Science, University College London, London WC1E 6BT, UK; 6 Department of Research and Education, University Hospital Zurich, 8091 Zurich, Switzerland

**Keywords:** arterial hypertension, epigenetics, microcirculation, endothelial cells, vascular smooth muscle cells, perivascular adipose tissue, shear stress

## Abstract

Systemic arterial hypertension (AH) is a multifaceted disease characterized by accelerated vascular aging and high cardiometabolic morbidity and mortality. Despite extensive work in the field, the pathogenesis of AH is still incompletely understood, and its treatment remains challenging. Recent evidence has shown a deep involvement of epigenetic signals in the regulation of transcriptional programs underpinning maladaptive vascular remodeling, sympathetic activation and cardiometabolic alterations, all factors predisposing to AH. After occurring, these epigenetic changes have a long-lasting effect on gene dysregulation and do not seem to be reversible upon intensive treatment or the control of cardiovascular risk factors. Among the factors involved in arterial hypertension, microvascular dysfunction plays a central role. This review will focus on the emerging role of epigenetic changes in hypertensive-related microvascular disease, including the different cell types and tissues (endothelial cells, vascular smooth muscle cells and perivascular adipose tissue) as well as the involvement of mechanical/hemodynamic factors, namely, shear stress.

## 1. Introduction: Arterial Hypertension, Epigenetics and Residual Cardiovascular Risk

Systemic arterial hypertension (AH) is among the most important healthcare problems of the 21st century [1], contributing to the current rise in the global cardiovascular disease burden. Worldwide mortality is driven by ischemic heart disease and stroke, which are major cardiovascular events tightly related to hypertension [1]. In addition, the prevalence of AH is expected to rise, fueling high-risk cardiovascular phenotypes and promoting the onset of heart failure [2]. By 2030, two-fifths of adults in the US will suffer from AH [3]; by 2050, only one-fourth of adults will be free of cardiovascular disease unless effective preventive strategies are implemented [4].

One of the main reasons for the above-mentioned disease burden is the increasing aging of the population. Indeed, significant advances in the treatment of acute illness have increased the average life expectancy [3]. Age is a major risk factor for AH [5] and has a significant impact on cardiovascular risk [3], especially when considering vascular age, a more reliable marker of biological age [6,7]. This is particularly evident in the microcirculation [8], which represents the barrier between internal organs and the external environment and thus the first stage where cardiovascular risk factors exert their deleterious effects [9].

AH accelerates microvascular aging [10,11,12] by promoting a low-grade pro-inflammatory pro-oxidative environment [13] that compromises microvascular homeostasis [14,15]. Importantly, even by removing or mitigating noxious triggers (i.e., the inflammatory milieu [13], the pressure overload and the shear stress) by pharmacological therapies, the damage to the vascular wall is not entirely reversed [16], predisposing to a cluster of comorbidities in the following years [17]. This persistent damage finds its molecular basis in epigenetic modifications that are able to derail the expression of genes implicated in vascular homeostasis. Epigenetic changes accumulated throughout life represent an important determinant of residual cardiovascular risk in hypertensive patients [18,19], a hallmark of cardiometabolic disease [20]. Despite the enormous improvement over the last few years [21], gold-standard therapies cannot address the epigenetic damage present at the microvascular level [22]. As a result, even after the intensive treatment of their disease, these patients still carry a higher cardiovascular risk than the general population.

To better understand the mechanisms underlying residual cardiovascular risk, we must focus on the earliest step of the healthy-to-disease transition, which has many shared pathways with the cardiometabolic disease spectrum. Indeed, the notion that time-dependent diseases are part of the same multi-faceted spectrum is relatively novel and supported by growing evidence [23,24]. As these pathways are all characterized by a common and early involvement of the microcirculation [8,9,10,11,12], it seems crucial to focus on it.

The present review provides an overview of the epigenetic mechanisms underlying microvascular damage and increased cardiovascular risk in the setting of AH. 

## 2. Epigenetic Crosstalk within the Microvascular Unit

Epigenetic changes might be classified into three main categories: (i) DNA chemical modifications (e.g., DNA methylation); (ii) histone tails post-translational modifications; (iii) gene expression regulation by noncoding RNAs (e.g., microRNAs (miRNAs), PIWI-interacting RNAs, endogenous short interfering RNAs, long noncoding RNAs) [25]. DNA methylation consists of the binding of a methyl group to the 5′ region of a cytosine of the cytosine–guanine dinucleotide (CpG), defined as a CpG island. CpG methylation functionally suppresses gene transcription and is mediated by DNA methyltransferases (DNMTs). In addition to DNA methylation, DNA hydroxymethylation (i.e., the binding of a methyl group to the 5′ cytosine of a CpG island) has recently been discovered to be an epigenetic marker involved in the methylation reprogramming. However, its precise biological meaning still needs further investigation [26,27]. Histone tails post-translational modifications include methylation, acetylation, ubiquitination and phosphorylation. They come as specific clustered patterns, allowing for the hyperexpression of genes by opening the chromatin, or vice versa. The main enzymes regulating these processes are histone acetyltransferases, deacetylases, methyltransferases and demethylases. While acetylation is, overall, a chromatin opening modification, the effect of methylation depends on the methylated residue and the number of methylations. Finally, noncoding RNAs are involved in transcriptional and post-transcriptional regulations. In particular, based on their size, they can be further classified into small noncoding RNA (<200 nucleotides), including miRNAs, PIWI-interacting RNAs and endogenous short interfering RNAs, and long noncoding RNAs (200–2000 nucleotides). Their potential pathogenetic role might indicate their targeting as a promising therapeutic strategy [22].

Emerging evidence indicates that changes in the epigenetic landscape are tissue/cell-specific [28]. Specific epigenetic signals within the same tissue, organ or functional unit (such as in the microcirculation) can promote phenotypic changes affecting the surrounding microenvironment. The microvasculature plays a complex and crucial role in maintaining tissue homeostasis, and a fine crosstalk among different tissues and organs regulates its function [29]. Microvessels are the primary stage where the exchange of substrates, gases and waste products between the bloodstream and the surrounding tissues occurs [30]. Moreover, microcirculation is also involved in the modulation of systemic and local responses, such as coagulation and inflammation. 

**Figure 1:**
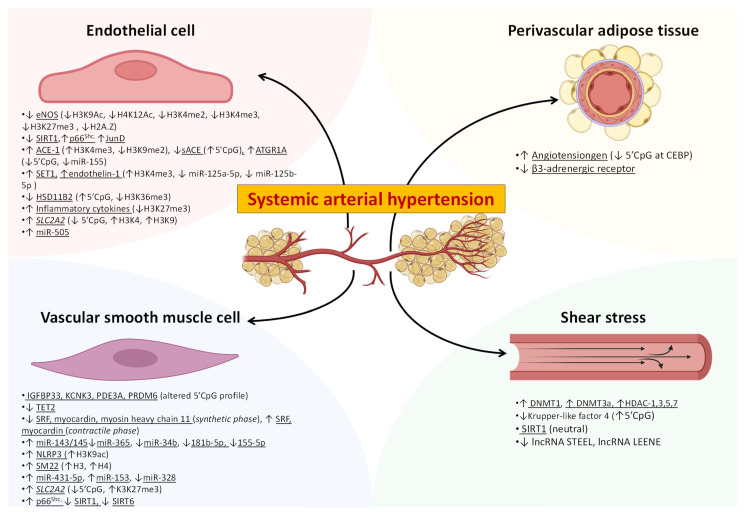
Epigenetic crosstalk of the microvascular unit in the context of hypertension. In systemic arterial hypertension, the four major elements of the microvascular unit (endothelial cells, vascular smooth muscle cells, perivascular adipose tissue and shear stress) possess a unique epigenetic signature that hinders the efficacy of the most recently developed drugs targeting hypertension. Future studies should address the complex network of epigenetic interaction and crosstalk underpinning the pathogenesis of systemic arterial hypertension. ↓: downregulated; ↑: upregulated11BHSD: 11β-hydroxysteroid dehydrogenase; ACE-1 angiotensin-converting enzyme-1: ATGR1A: angiotensin II receptor type 1a; CEBP: CCAAT/enhancer binding protein; eNOS: endothelial nitric oxide synthase; KCNK3: potassium two-pore domain channel subfamily K member 3; lncRNA: long noncoding RNA; PDE3A: phosphodiesterase 3A; PRDM6: PR domain-containing protein 6; sACE: somatic-ACE; SIRT1: silent information regulator 1; SLC2A2: Solute Carrier Family 2 Member 2; SM22: smooth muscle cell 22; SRF: serum response factor.

The major elements concurring to maintain local homeostasis are endothelial cells (ECs), vascular smooth muscle cells (VSMCs) and perivascular adipose tissue (PVAT). In addition, the constant wall shear stress exerted by the bloodstream, paired with the ECs mechano-transduction capacity, is also involved in the maintenance of the homeostatic balance. Altogether, these distinct components constitute the functional microvascular unit [30]. Figure 1 summarizes the most important epigenetic modifications occurring within the microvascular unit in AH. 

### 2.1. Endothelial Cells

ECs play a wide range of different functions within the microvasculature. They regulate the vascular tone by virtue of the dynamic crosstalk with VSMCs, PVAT and renin-angiotensin-aldosterone (RAAS) system membrane receptors and neuronal terminations. ECs also transduce mechanical stimuli from the local bloodstream flow and can act as nutrient sensors and mediators of insulin signaling. Moreover, they have been reported to be promoters and perpetrators of the microvascular inflammatory response, impaired angiogenesis, the modulation of salt sensitivity and the response to sex hormones. The centrality of the vascular endothelium in all these functions [31] makes it an essential actor in the healthy-to-disease transition and has placed it at the center of vascular research over the last forty years [32]. The endothelium is characterized by an array of different epigenetic signals that fine-tune the transcriptome, thus securing cellular identity and function. For example, specific DNA and histone methylation patterns define an active vs. quiescent endothelium. These two endothelial phenotypes change during life but might be simultaneously present within the same organ or tissue and even within the same microvessel. This can be observed by quantifying the different expression of the von Willebrand Factor (vWF) among neighboring endothelial cells from the same vascular bed. Whilst, during life, in some tissues, vWF expression is either low (e.g., liver sinusoidal) or constant (e.g., aorta), indicating a more quiescent endothelium, in others (e.g., heart, skeletal muscle, brain), the expression is higher and very variable, thus suggesting endothelial activation. This dynamic epigenetic mosaicism represents the molecular basis of adaptive endothelial homeostasis. It is regulated by a DNA methylation of CpG islets of the vWF promoter, which occurs randomly and dynamically during the lifetime, allowing for a faster response time and adaptation to biological stimuli [33]. 

#### 2.1.1. Endothelial Function

Many of these functions are dependent on the capacity of the endothelium to generate nitric oxide (NO)—a crucial mediator of ECs function and homeostasis—by endothelial nitric oxide synthase (eNOS). Reduced NO availability is the hallmark of endothelial dysfunction [8]. The eNOS promoter exhibits a unique histone code characterized by the enrichment of active epigenetic signatures, including H3K9Ac, H4K12Ac, H3K4me2, H3K4me3, H3K27me3 and the H2A.Z histone variants in ECs [34]. In AH, this code is altered and leads to an impairment in eNOS expression and activity [35]. Endothelial-dependent relaxation impairment in AH, as well as in other cardiometabolic diseases [16], is also mediated by epigenetic pathways involving the adaptor p66Shc [36] and JunD [37,38]. In hypertensive rats, resveratrol, a non-selective silent information regulator 1 (SIRT1) activator [25], is associated with the restored methylation of H3K27me3 on the eNOS promoter [39] rescuing endothelial function, as also observed in human models of cardiometabolic disease [24]. 

#### 2.1.2. Endothelial Response to the Neurohormonal Environment

The endothelium also modulates the response of the vessels to neurohormonal stimuli. Changes in the epigenetic landscape play an essential role in this respect. The enrichment of H3K4me3 and decreased H3K9me2 levels were found at the angiotensin-converting enzyme (ACE)-1 promoter, which is associated with ACE-1 upregulation [40]. In vitro and in vivo experiments have shown that AH leads to the hypermethylation of ACE-1 promoter/reporter constructs of the somatic ACE (sACE) promoter, downregulating its expression and reducing its transcriptional activity [30]. The angiotensin II receptor type 1a gene (*ATGR1A*) promoter in ECs from the aorta and mesenteric arteries of SHR rats shows progressive hypomethylation with age compared to wild-type animals [41]. Human endothelial cells isolated from the placenta of pre-eclamptic women show higher miR-155 levels [42] as a compensatory mechanism for reducing the levels of angiotensin II receptor type 1 (*AGTR1*) mRNA. This miRNA targets the polymorphic sequence in the 3′UTR of *AGTR1* mRNA [43]. 

Additionally, angiotensin II recruits SET1, a histone H3K4 tri-methyltransferase, to the promoter of endothelin-1 [44] by activating protein 1 (AP1) to methylate H3K4. This increases the expression of endothelin-1 (ET-1), leading to uncontrolled AH and subsequent organ damage, namely, cardiac hypertrophy. miR-125a-5p and miR-125b-5p were shown to downregulate ET-1 expression in ECs, in line with their reduced level in hypertensive rats [45]. In normal conditions, aldosterone regulates sodium reabsorption due to the inactivation of cortisol to cortisone by 11β-hydroxysteroid dehydrogenase (*HSD11B2*). In AH, the ECs *HSD11B2* gene promoter is hypermethylated [17], while its H3K36me3 levels are reduced [46]. This leads to a tetrahydrocortisol/tetrahydrocortisone ratio, which is also a hallmark of AH [47]. 

#### 2.1.3. Endothelial Modulation of the Inflammatory Response and Salt Sensitivity

The inflammatory response is also filtered by the ECs’ capacity to modulate or activate it firsthand. Inflammatory stimuli such as lipopolysaccharide (LPS) treatment promote the demethylation of H3K27me3 on the promoter of inflammatory genes by activating the demethylase JMDJ3 [48]. The inflammatory response provoked by S-adenosylhomocysteine by NF-κB also collaterally downregulates the methyltransferase EZH, thus reducing H3K27 trimethylation [49]. 

In ECs, a high-salt diet promotes the hypomethylation of the Solute Carrier Family 2 Member 2 (*SLC2A2*) gene, with subsequent higher levels of membrane transporter Na^+^-K^+^-2Cl-cotransporter 1 (NKCC1) [50] and the downregulation of histone lysine-specific demethylase 1 (LSD1), leading to an increased methylation of histone H3K4 or H3K9 [42]. Additionally, the hypertensive signature leads to impaired angiogenesis by upregulating miR-505 in ECs [51].

### 2.2. Vascular Smooth Muscle Cells

VSMCs function is tightly dependent on ECs. However, preserving VSMCs homeostasis is crucial, as their level of contraction/relaxation determines the vasomotor tone, with subsequent changes in the lumen diameter and arterial resistance. In the microcirculation, VSMCs contract in response to blood pressure variations within the physiological range. Their myogenic activity has both a phasic and static component. While the first is involved in the acute response to stimuli, the latter maintains the flow in homeostatic conditions. In AH, both components are impaired [52]. Another major VSMCs feature lies in their high plasticity. In healthy conditions, they display a fully mature contractile phenotype characterized by low proliferation and a hyperexpression of contractile proteins. In response to noxious stimuli, they can de-differentiate to a synthetic phenotype, characterized by the production of the extracellular matrix, proliferation, migration and angiogenesis. When sustained, it leads to inward hypertrophic remodeling [53], a hallmark of AH [54]. 

#### 2.2.1. Modulation of VSMCs Plasticity

High blood pressure induces epigenetic changes that foster the de-differentiation and the consequent proliferative activity of VSMCs. A recent trans-ancestry genome-wide association study (GWAS) showed that different blood pressure phenotypes display opposite methylation levels in four genes involved in vascular tone and VSMC plasticity: insulin-like growth factor binding protein 3, potassium two-pore domain channel subfamily K member 3, phosphodiesterase 3A and PR domain-containing protein 6 [55]. AH was also associated with the downregulation of ten-eleven translocation-2 (TET2) [56]. TET2 is responsible for cytosine 5-hydroxymethylation on the promoters of myocardin, serum response factor (SRF) [57] and myosin heavy chain 11 [56], which are all involved in the contractile VSMCs phenotype. SRF and myocardin are also involved in the tight control of proliferation by upregulating the miR-143/145 cluster [58]. Mice lacking both miR-143 and miR-145 have reduced blood pressure but are more prone to vascular injury [59]. Although the reduced blood pressure might reflect the laxity of the vessel wall, reduced levels of miR-143 are also associated with contractile dysfunction in skeletal muscle cells from older mice [60]: the microcirculation structure and function might thus be directly affected by the VSMCs down-regulation of the miR-143/145 axis in age-related diseases such as AH. However, further studies are needed to elucidate miR-143/145-specific contribution in this context. Higher angiotensin II levels in AH decreased miR-365, increasing VSMC proliferation [61]. Similarly, miR-34b levels are reduced in spontaneously hypertensive rats, leading to a higher VSMCs proliferation rate [62]. On the other hand, the upregulation of miR-181b-5p prevents angiotensin II-induced VSMCs proliferation [63], while the upregulation of miR-155-5p inhibits VSMCs proliferation via suppressing angiotensin-converting enzyme expression [64]. However, persistently high levels of angiotensin II will eventually lead to the downregulation of miR-181b-5p and miR-155-5p, which are critical regulators of the VSMCs phenotype. VSMCs are also involved in hypertension-related microvascular inflammation, which, in turn, impairs VSMCs functionality. In hypertensive rats, the histone acetylases EP300-binding protein and CREB-binding protein increase the H3K9ac in the NLRP3 promoter. Its activation promotes the hyperactivation of the NF-κB pathway, ultimately resulting in VSMC remodeling, transformation and proliferation [65].

#### 2.2.2. VSMCs Contractility

A VSMCs-altered contractile response is also a hallmark of AH. Whilst, in normal conditions, TET2 modulates the expression of SRF, allowing for the VSMCs between synthetic and contractile states, the hypertension-related downmodulation of TET2 [56] leads to a reduced response to vascular injury, and when VSMCs switch to the contractile state, this leads to the hyperacetylation of H3 and H4 on the smooth muscle cell 22 (*SM22*) [66] and myocardin genes by SRF. This induces increased vascular stiffness, a hallmark of AH [67]. VSMCs also overexpress miR-431-5p, worsening vascular stiffening [68]. In murine models, miR-153 is elevated and targets potassium voltage-gated channels, increasing the myogenic basal tone [69]. On the other hand, miR-328 is downregulated in VSMCs, and the loss of inhibition on the L-type voltage-gated calcium channel activity promotes AH. Peculiarly, aging upregulates miR-328 in the same murine model, pointing out its hypertension-specificity [70]. A high-salt diet leads to the hypomethylation of the SLC2A2 gene, increasing NKCC1 also in VSMCs [50]. Angiotensin also upregulates NKCC1, increasing H3Ac and decreasing H3K27me3 [71]. Moreover, the adaptor p66Shc restricts the activation of transient receptor potential cation channels, downregulating Ca2+ influx [72] and reducing spontaneous Ca2+ oscillations [73] on VSMCs. 

#### 2.2.3. VSMCs Senescence

Finally, several mechanisms implicated in VSMCs homeostasis are progressively impaired in AH. The overexpression of specific sirtuins, a histone deacetylase family, such as SIRT1 and SIRT6, inhibits VSMCs proliferation and extracellular matrix synthesis in hypertensive rats [74] and protects from vascular senescence by reducing telomere H3K9 acetylation [75], respectively.

### 2.3. Perivascular Adipose Tissue

Perivascular adipose tissue (PVAT) is crucial in maintaining microcirculatory homeostasis. First, it modulates the inflammatory response thanks to its paracrine and juxtracrine capacities. PVAT-secreted molecules (i.e., adipokines) such as tumor necrosis factor-α (TNF-α), interleukin-6 (IL-6), monocyte chemoattractant protein-1, leptin as well as angiotensinogen [76] directly affect the microvascular function. They modulate the inflammatory pathways [44], the metabolic response to external stimuli such as increased blood pressure or nutrient overload and also the microvascular contractility [76]. Moreover, in healthy subjects, PVAT has a protective anti-contractile property and a protective brown phenotype whose origin is still debated [77]. These features are lost in AH [78]. However, there is a lack of a complete understanding of the involvement of PVAT in the cellular crosstalk in the microvascular unit in conditions of health and disease, and evidence is sometimes difficult to interpret [79]. The molecular mechanisms of its epigenetic signatures under hypertensive damage are almost uncharted territory. In rats, a high-salt diet induces hypomethylation around two CCAAT/enhancer binding protein (CEBP) binding sites and a transcription start site, ultimately leading to upregulated angiotensinogen gene expression in PVAT [80]. Additionally, in the visceral adipose tissue of obese hypertensive patients, the β3-adrenergic receptor was found to be hypermethylated [81].

### 2.4. Shear Stress

Although it might seem difficult to imagine the bloodstream flow as a homeostatic modulator, the pressure exerted on the microvascular wall, particularly its frictional component, i.e., the shear stress, impacts the functionality of the microvascular unit. Indeed, endothelial cells are professional flow sensors, and the mechanical signal exerted on them is transduced to all the actors involved in microvascular homeostasis. Indeed, physiologic shear stress exerts a protective function on the vascular wall in healthy conditions by regulating the expression of protective genes through mechanotransduction. On the other hand, in hypertensive subjects, its alterations lead to the hyperexpression or downregulation of specific elements, often by epigenetic means [82]. Different types of flows (e.g., laminar vs. disturbed) induce different methylation patterns in endothelial cells [83]. The adaptor protein p66Shc was shown to be implicated in endothelial damage by promoting a pro-oxidative environment after the cyclic stretch to the vascular wall [84]. 

Disturbed flow, as observed in the mouse after the partial ligation of the carotid artery, led to upregulation of the DNMT1. In HUVECs, oscillatory shear stress similarly led to DNMT1 upregulation, and the inhibition of DNMT1 prevents the monocyte adhesion that is a consequence of oscillatory shear stress [85]. Disturbed flow also leads to the hyperactivation of DNMT3A, leading to the hypermethylation of the Krupper-like factor 4 promoter, an anti-inflammatory and anti-thrombotic protein also involved in the modulation of eNOS signaling [86]. Histone deacetylase 3 in endothelial cells is also enhanced by a disturbed flow [87]. Similarly, it promotes the expression and the accumulation of histone deacetylases 1, 3, 5 and 7 [88], while it does not affect the expression of SIRT1, which is increased during normal pulsatile flow [89]. Finally, a disturbed flow regulates the expression of specific long noncoding RNA such as STEEL [90] and LEENE, which are both enhancers of eNOS function that are increased during pulsatile flow but decreased during oscillatory flow [91].

### 2.5. Other Actors Involved in Microvascular Control 

Though this review will not extensively discuss their role, neural terminations and inflammatory cells are involved in the microcirculatory crosstalk. Indeed, the theory of the neurogenic component of AH strongly supports a direct epigenetic crosstalk between the central and the autonomic nervous systems and inflammatory elements [92]. However, their epigenetic cues have yet to be investigated accurately. On the other hand, there is evidence about the epigenetic networks involved in the transcriptional regulation of inflammation in different vascular cells in AH. Euchromatic histone-lysine n-methyltransferase 2, a methyltransferase specifically overexpressed in leukocytes in healthy conditions, is hypomethylated in hypertensive patients, as reported by a longitudinal GWAS [93]. Peripheral blood mononuclear cells from patients with AH have a specific miRNA profile, showing lower levels of miR-133, miR-143 and miR-145 and higher levels of miR-1 and miR-21 [94]. In macrophages, the deacetylation of NLRP3 by the overexpression of SIRT2 NAD+-dependent deacetylase represses inflammasome activation, preventing chronic inflammation [95] and suggesting its protective role in the context of cardiometabolic diseases such as AH. Finally, telocytes deserve to be mentioned. As an interstitial cell type found in various organs of the human body, including the microvasculature, they have a specific role in the context of the cardiovascular system, regulating the cardiac and vascular development, structure and tone [96]. Although their function in the microcirculatory system has yet to be elucidated, they have been recently shown to be involved in crosstalk with ECs [97] mediated by mi-21-5p signaling to improve the angiogenetic response after tissue injury [98]. Additionally, they are spatially related to VSMCs and contribute to regulating the vascular tone in different body districts, from the peripheral to the cardiac and cerebral microcirculation [98,99]. When their physiologic function is lost, they are responsible for derangement in the vascular wall homeostasis, contributing to an increased susceptibility to vasospasm, which is another determinant in the pathogenesis of AH [100]. However, direct evidence of their role and, in particular, their epigenetic signature in the context of microcirculation in AH needs to be adequately investigated by further studies.

## 3. Perpetrators of Damage: Oxidative Stress and Early-Life Stress

In AH, epigenetic damage on the microvascular wall is ultimately inflicted by increased oxidative stress [101]. Reactive oxygen species (ROS) promote histone modification and DNA methylation by strand breaks, cytosine methylation, hydroxymethylation or 8-oxo-2-deoxyguanosine formation [102]. Additionally, they alter the expression of miRNAs and long noncoding RNAs [103]. Through oxidative stress [14], AH exerts its epigenetic damage, which persists even after the noxious stimulus (e.g., RAAS hyperactivation) is removed or controlled by the intensive control of risk factors. Moreover, the deep interaction between the environment and epigenetics [25,104] primes to the development of AH many years before its onset. Chronic adult-onset diseases can originate from exposure to a pro-oxidant environment as early as during prenatal life, which has led to the formulation of a theory known as the developmental origins of health and disease [105]. Partially related to oxidative stress, genotoxic stress might also play a role in the onset of AH, as has been suggested in renovascular AH [106]. Environmental and chemical genotoxic agents, such as arsenic, can alter the epigenetic pathways involved in AH, modifying the DNA methylome and histone methylome and acetylome [107]. However, there is little evidence of specific genotoxic microcirculatory damage associated with AH. Genotoxic anticancer drugs such as docetaxel, doxorubicin and cyclophosphamide, already extensively studied in heart failure [108], might also be critical in the development of AH, inducing microvascular dysfunction mediated by the demethylation of the NAPDH-oxidase 4 promoter [109].

Early-life stress has major relevance to the epigenetic signature of AH. A low birth weight, decreased infant and childhood growth, a low adult body mass index and decreased maternal weight and nutrition all define an epigenetic signature in newborns, as well as maternal exposure to noxious environmental stimuli [110]. In rats, pregnant mothers exposed to higher levels of IL-6 give birth to offspring which develop, starting from the fifth week, alterations in RAAS signaling, leading to AH and vascular remodeling [111]. Similarly, prenatal exposure to LPS results in AH in the offspring [112] by increasing histone H3 acetylation (H3AC) on the ACE1 promoter. Prenatal treatment with ascorbic acid, a strong antioxidant, preserves the phenotype by inducing histone deacetylation of the ACE1 promoter [113]. Resveratrol prevents, in male offspring, AH development induced by the combination of a maternal high-fat diet (delivered during gestation and lactation) and an offspring postweaning high-fat diet [114]. Parental vitamin D deficiency leads to hypermethylation of the promoter region of the pannexin-1 gene in rats, leading to AH from an early age [115]. The early exposure to stimuli leading to AH also shows sex differences that deserve further investigation. In rats, mothers treated with L-NAME, dexamethasone and a high-fructose diet since the first gestational day give birth to male offspring that will develop AH in adulthood [116]. Maternal eNOS deficiency leads to an alteration of blood pressure control in the offspring [117], while paternal eNOS deficiency only affects glucose homeostasis [118]. As this evidence leaves many open questions, further studies in this direction are needed to grasp an accurate understanding of the developmental origin of AH. 

## 4. Targeting Epigenetics

Although the evidence on the epigenetic signature of systemic AH is steadily growing, a knowledge gap hinders its clinical translation, as evidenced by the limited number of studies targeting epigenetics in AH (Table 1). Though several epigenetics drugs are being proposed and tested, such as vorinostat [119], trichostatin A [120] and specific anti-miRNAs [22], only a few have already been proposed for human use. The most promising ones have been developed to target pathways of damage, such as vascular inflammation and angiogenesis, shared by most cardiometabolic diseases. In this sense, a pan-inhibitor of vascular inflammation, RVX-208, has already undergone a phase III clinical trial with promising results [121,122,123,124], targeting pathways indirectly related to AH [125]. Sirtuins are also being explored as potential rescuers of the many detrimental functions described above [24,126,127,128,129]. In a human cohort, a long-term high dietary intake of NAD+ precursors (which ultimately stimulate SIRT1) was associated with a lower blood pressure and reduced CVD risk [130]. 

However, if we look at epigenetic drugs for AH, no drug targeting specific hypertensive-related epigenetic pathways has been developed and satisfactorily tested in humans. Additionally, epigenetic drugs often fail to achieve successful tissue delivery and tissue specificity, which might also imply toxicity due to off-target effects [129]. In a complex setting such as the microvasculature, this might lead to higher therapeutic unsuccess rates.

At present, the non-pharmacologic approaches, namely, calorie restriction and weight loss, seem more promising in terms of immediate translation. Calorie restriction is one of the most powerful interventions for preserving cardiometabolic health [131], as it promotes ideal microvascular homeostasis through epigenetic reprogramming [12]. Severely obese patients showed reduced inflammatory indices paired with an improved small arteries endothelial function after a short (3 weeks), very-low-calorie diet [132]. Obese patients, after eight weeks of moderate calorie restriction, showed reduced C-reactive protein, TNF-α, IL-6 and endothelium-derived ROS [133]. Long-term studies such as the phase II CALERIE trial also reported encouraging results, demonstrating an amelioration of the whole cardiometabolic profile [134]. Calorie restriction is often paired with weight loss, thus showing powerful effects in cardiometabolic homeostasis improvement [135]. Weight loss, especially when marked and sustained, is a strong epigenetic modulator [136]. Particular interest was recently raised by the DiRECT trial, which demonstrated, for the first time, the possibility of achieving type 2 diabetes remission [137]. Bariatric surgery is one of the most successful treatments for total weight loss [138], with no substantial negative effect compared to dietary strategies [139] and a very positive impact on cardiometabolic homeostasis; it is now also being defined as “metabolic surgery”. Recently, the GATEWAY (Gastric Bypass to Treat Obese Patients With Steady Hypertension) trial showed how bariatric surgery might lead to the remission of AH [140]. Metabolic surgery-obtained weight loss induces epigenetic remodeling on PVAT, restoring its vasorelaxant function [141]. A recent meta-analysis [142] showed that a specific dietary approach defined as DASH (Dietary Approach to Stop Hypertension) [143] and the combination of aerobic exercise and isometric training, low-sodium and high-potassium salt, comprehensive lifestyle modification, breathing control and meditation are among the most effective non-pharmacological treatments for stopping the progression from pre-hypertension to AH. 

While the advantage of these studies is that their results are directly transferable into clinical practice, they present at least two main disadvantages. First, they often do not focus exclusively on the hypertensive phenotype but rather on patients who are obese or overweight. They therefore do not allow the epigenetics pathways specifically linked to AH to be identified. Second, there is a profound lack of knowledge of the mechanisms underlying their beneficial effect, especially regarding their impact on epigenetics remodeling in the microvasculature. The changes in chromatin remodeling underpinning the positive outcome data seen with weight loss and calorie restriction deserve further exploration in the near future.

**Table 1:** Human evidence (direct or indirect) of targeting the epigenetic signature of arterial hypertension.

Intervention	Study Type	Study Population	Effect	Ref
Pharmacological strategies				
RVX-208	RCT	CAD, T2D and low HDL	↓ 18% RR of MACE (*p* = ns) ↓ 53% RR of HHF (*p* = 0.01) ↓ 28% RR of CVD + HHF (*p* = 0.04)	[121,124]
	RCT	CAD, T2D, low HDL and CKD	↓ 50% RR of MACE (*p* = 0.04) ↓ 52% RR of HHF (*p* = 0.04)	[122]
		CAD, T2D, low HDL without CKD	↓ 4% RR of MACE (*p* = ns) ↓ 24% RR of HHF (*p* = ns)	
	RCT	CAD, T2D, HDL, MoCA ≤ 21	↑ MoCA (+3 points; *p* = 0.02)	[123]
SIRT1	Translational (ex vivo)	Older adults with obesity	↑ 21% microvascular function (*p* < 0.01)	[24]
Resveratrol	RCT	Overweight and obese Overweight and obese	No effect on glucometabolic profile No effect on FMD	[127,128]
	Meta-analysis	Cardiometabolic patients	↑ 77% FMD; no effect on SBP/DBP	[129]
Non-pharmacological strategies				
High NAD+ intake	Cohort	Age- and sex-matched random sample	↓ 11–14% RR of ACM (*p* = 0.009–0.038) ↓ 24–25% RR of CVD (*p* = 0.008–0.015) ↓ 1.57–1.66 mmHg SBP (*p* = 0.009–0.013) ↓ 0.56–0.58 mmHg DBP (*p* = 0.053–0.046)	[130]
VLCD	Prospective	Patients with severe obesity	↑ 8% saRHI (*p* = 0.015) ↓ 4.5% SBP (*p* = 0.049)	[132]
300–500 kcal CRD	Prospective	Patients with obesity	↓ 6 mmHg SBP (*p* < 0.01)	[133]
25% CRD	RCT	Young and middle-aged healthy non-obese	↓ 4.35 mmHg SBP (*p* < 0.01) ↓ 4.85 mmHg DBP (*p* < 0.01)	[134]
Bariatric surgery	Randomized non-blinded trial	Patients with severe obesity and hypertension	↓ 30% anti-hypertensive drugs (*p* < 0.01) 45.8–51% hypertension remission	[140]
Bariatric surgery	Translational (ex vivo)	Patients with severe obesity	↑ PVAT anticontractile effect (*p* < 0.01)	[141]
Lifestyle changes *	Meta-analysis	Patients with pre-hypertension or hypertension	↓ 6.97 mmHg SBP (*p* < 0.01) ↓ 3.54 mmHg DBP (*p* < 0.01)	[142,143]

* Among all interventions, a specific dietary approach defined as DASH (Dietary Approach to Stop Hypertension) or the combination of aerobic exercise and isometric training, low-sodium and high-potassium salt, comprehensive lifestyle modification, breathing control and meditation were the most effective. ↑: increase; ↓: reduction; ACM: All-cause mortality; CAD: coronary artery disease; CKD: chronic kidney disease; CRD: calorie reduction diet; CVD: cardiovascular death; DBP: diastolic blood pressure; FMD: flow-mediated dilation; HDL: high-density lipoprotein; HHF: hospitalization for heart failure; MACE: Major Adverse Cardiovascular Events; MoCA: Montreal Cognitive Assessment; NAD^+^: nicotinamide adenine dinucleotide; PAD: Peripheral Artery Disease; PVAT: perivascular adipose tissue; RCT: randomized controlled trial; RR: risk reduction; saRHI: small artery reactivity to postischemic hyperemia index; SBP: systolic blood pressure; T2D: type 2 diabetes; VLCD: Very Low-Calorie Diet.

## 5. The Next Steps Ahead

Systemic arterial hypertension is a multifactorial disease characterized by a multifaceted epigenetic signature involving a profound crosstalk between the different elements of the microvascular unit. What is certain is that future studies should expand the current knowledge, thus unravelling novel molecular targets and personalized therapies in this setting. Recently explored pathways are drawing attention, such as the Hippo one, involved in VSMCs and endothelium signaling [144]. The methylation of its effector YAP has been found to be dysregulated in many cardiometabolic diseases. It fosters oxidative stress-mediated cardiac damage following ischemia, albeit its exploration in hypertensive models is limited to a few observations [145]. On the other hand, sortilin, a molecule involved in the acid sphingomyelinase pathway, has been recently discovered as a potential pathogenetic factor in hypertensive subjects. However, its epigenetic regulation still needs to be explored [146]. 

The relevance of these investigations lies in the possibility of clinical translation. For example, by leveraging CRISPR-Cas9 editing [147], we might eventually overcome problems related to tissue delivery, cell-specificity and off-target effects. Yet, we would still miss fully understanding the molecular cues involved. Unravelling the epigenetic bases involved in the early stage of damage in humans means paving the way for effective drug development strategies targeting specific epigenetic pathways involved in hypertension.

## Data Availability

Not applicable.
